# Optimized Design and Preparation of Ag Nanoparticle Multilayer SERS Substrates with Excellent Sensing Performance

**DOI:** 10.3390/bios13010052

**Published:** 2022-12-29

**Authors:** Ping Wen, Feng Yang, Xiaoling Hu, Yi Xu, Shu Wan, Li Chen

**Affiliations:** 1 Key Laboratory of Optoelectronic Technology and Systems, Ministry of Education, Key Disciplines Lab of Novel Micro-Nano Devices and System Technology, College of Optoelectronic Engineering, Chongqing University, Chongqing 400044, China; 2 School of Intelligent Manufacturing, Sichuan University of Arts and Science, Dazhou 635000, China; 3 School of Artificial Intelligence, Chongqing Technology and Business University, Chongqing 400067, China; 4 The Water Quality Monitoring Network of National Urban Water Supply Monitoring Station of Chongqing, Chongqing 400074, China

**Keywords:** SERS, local electric field (LEF), Ag nanoparticle (AgNP) multilayer, propagating surface plasmons (PSPs)

## Abstract

Nanoparticle multilayer substrates usually exhibit excellent SERS activity due to multi-dimensional plasmon coupling. However, simply increasing the layers will lead to several problems, such as complex manufacturing procedures, reduced uniformity and poor reproducibility. In this paper, the local electric field (LEF) characteristics of a Ag nanoparticle (AgNP) multilayer were systematically studied through finite element simulations. We found that, on the glass support, the LEF intensity improved with the increase in the layers of AgNPs. However, the maximum LEF could be obtained with only two layers of AgNPs on the Au film support, and it was much stronger than the optimal value of the former. To verify the simulation results, we have successfully prepared one to four layers of AgNPs on both supports with a liquid–liquid interface self-assembly method, and carried out a series of SERS measurements. The experimental results were in good agreement with the simulations. Finally, the optimized SERS substrate, the 2-AgNP@Au film, showed an ultra-high SERS sensitivity, along with an excellent signal uniformity, which had a detection ability of 1 × 10^−15^ M for the Rhodamine 6G (R6G) and a relative standard deviation (RSD) of 11% for the signal intensity. Our study provides important theoretical guidance and a technical basis for the optimized design and application of high-performance SERS substrates.

## 1. Introduction

Surface-enhanced Raman spectroscopy (SERS) relies mainly on the local electric field (LEF) hotspots produced by surface plasmons in a noble metallic nanostructure to enhance the weak Raman scattering signal [1,2,3,4]. Thus, rational design and manufacturing of SERS substrates are very important for obtaining SERS signals with excellent sensitivity, uniformity, and reproducibility [5,6,7,8]. Noble metal nanoparticle arrays support high-quality plasmon resonance, which can greatly enhance the LEF intensity at nanogaps, and provide high Raman enhancement factors (EF), thus attracting widespread attention [3,9]. For example, Zhao and co-workers [10] assembled a closely adjacent AgNP monolayer on polydimethylsiloxane to the in situ SERS detection of pesticide residues. Teng et al. [11] prepared a AgNP monolayer SERS substrate with the self-assembly of AgNPs on a water/oil interface, and realized the trace SERS detection of enrofloxacin. Compared with the monolayer SERS substrate, the multilayer SERS substrate could form a greater number of and stronger LEF hotspots due to the plasma coupling in both horizontal and vertical directions [12]. Meanwhile, a three-dimensional structure often provides a larger surface area to adsorb more target molecules [13,14]. Therefore, a greater number of SERS signals are typically collected on multilayer SERS substrates. For example, Oh et al. [15] prepared an Au nanosphere multilayer SERS substrate on a glass support, and their research showed that the SERS intensity gradually improved with the increase in layers, although it gradually stabilizes when the number of layers exceeds four. Lin et al. [14] prepared a Ag nanocube multilayer SERS substrate with the self-assembly method, and their research results were similar to those of Oh et al., i.e., the SERS intensity increased with the increase in layers, while the Raman enhancement factor tends to be stable when it exceeds four layers.

Although hotspots with a high LEF intensity could be provided by stacking more particle layers, there were still some problems to resolve here. First, the uniformity and repeatability of the SERS substrate will worsen by simply adding more nanoparticle layers [9,16]. Second, a higher LEF intensity does not guarantee a stronger signal in the actual SERS measurement. Zhang et al. [17] showed that when the layers exceed two, the SERS signal of the lower layer greatly weakened due to the shielding and scattering of the upper layer particles. Meanwhile, Liu et al. [18] also found that the target molecules had difficulty entering the bottom hotspot region due to the steric hindrance; as a result, the Raman signal could not be effectively enhanced. Therefore, under the premise of ensuring high LEF intensity, fewer particle layers are of great significance to improve SERS efficiency.

Herein, the LEF distribution and characteristics of the AgNP multilayer on Au film and glass were systematically studied with finite element simulations. The results showed that the intensity, variation trends, and distribution position of the maximum LEF intensity of the AgNP multilayer on the glass were completely different from those of the AgNP multilayer on the Au film. Due to the hybridization between the propagating surface plasmons of the Au film and the localized surface plasmons of the AgNPs, the maximum LEF could only be obtained by stacking two layers of AgNPs on the Au film, which was much stronger than the optimal value on the glass support. Then, to verify the simulation results, one to four layers of AgNPs were prepared on both supports with a liquid–liquid interface self-assembly method, a series of SERS experiments were carried out, and the experimental results were in good agreement with the simulations. Finally, the optimized SERS substrate, i.e., the 2-AgNP@Au film, shows ultra-high SERS activity, along with excellent signal uniformity, which had a limit of detection (LOD) of 1 × 10^−15^ M for the R6G and a relative standard deviation (RSD) of 11% for the signal intensity. Thus, a surprising SERS sensitivity can be obtained with just two layers of AgNPs, and the problems caused by simply increasing the layers were effectively overcome. Our study could provide important theoretical guidance and a technical basis for the optimized design and application of high-performance SERS substrates.

## 2. Materials and Methods

### 2.1. Materials and Reagents

Silver nitrate (AgNO_3_, 99.7%), trisodium citrate (Na_3_C_6_H_5_O_7_·2H_2_O, AR), n-hexane (C_6_H_14_, AR), ethanol (C_2_H_6_O, 99.9%), and rhodamine 6G (R6G, AR) were acquired from Chongqing Chemical Reagent Company Ltd. (Chongqing, China). Silicon wafers with 〈100〉 crystal orientations were purchased from Suzhou Jingxi Electronic Technology Co., Ltd. (Suzhou, China). All glassware used in the experiments was washed with a piranha solution, then thoroughly rinsed with ultrapure water and dried before use.

### 2.2. Modeling and Simulation

The COMSOL Multiphysics tool was used to simulate the LEF distribution of AgNPs. The excitation light with a wavelength of 532 nm and an intensity of 1 V/m was incident along the *z*-axis and polarized along the *x*-axis, so that the near-field coupling between adjacent AgNPs along the *y*-axis was weak and negligible [17]. Therefore, the AgNP monolayer and multilayer were simulated as periodic structures along the *x*-axis, and only one particle was considered along the *y*-axis. Periodic boundaries were used on the *x*-axis to simulate a periodic nanoparticle array. Perfectly matched layer boundary conditions are used on the y and z axes to exclude nonphysical reflections. The dielectric function of Au and Ag was taken from the literature [19,20]. In order to save computing resources and computing time, a non-uniform mesh is used to discretize the dimensional space. Geometry in this work comprises a Ag nanoparticle array located on 120 nm thick Au film. The schematic diagram of simulation is shown in Figure S1 (in Supplementary Materials). The particle size and the gap size were set as 50 nm and 4 nm, respectively, which were determined according to the characterization results (Figure S2, Supplementary Materials).

### 2.3. Preparation of AgNP Multilayer SERS Substrates

The AgNPs with an average diameter of 50 nm (Figure S2, Supplementary Materials) were prepared using the method proposed by Lee and Meisel [21]. A AgNO_3_ solution (100 mL, 1 mM) was continuously stirred and heated to boiling, after which a sodium citrate solution (1.8 mL, 1 wt%) was quickly added with continuous stirring and heating for at least 60 min. During this process, the color of the solution gradually changed from colorless to bright yellow, with a final change to yellow-green, after which the color did not change, indicating that the preparation of the AgNPs was successful. After filtering the prepared Ag sol through a 0.1 μm pore size membrane, it was poured into a brown bottle and stored in a refrigerator at 4 °C.

The SERS substrate was prepared by the liquid–liquid interface self-assembly method [22,23] and the detailed experimental steps are as follows. (1) A 120 nm thick Au film was deposited on an Si wafer using magnetron sputtering. The Au-plated Si wafer and glass were cut into small pieces with dimensions of 1 × 1 cm^2^ and all the pieces were placed in a piranha solution and heated for 30 min to remove any organic contaminants, then thoroughly rinsed with ultrapure water and dried with nitrogen. (2) We concentrated 1/3 of the freshly prepared Ag colloids (centrifuge at 7000 rpm for 5 min, remove the supernatant and then disperse it in ultrapure water). (3) The concentrated Ag colloids were mixed with n-hexane, vortexed intensely until a bright AgNP monolayer was formed at the oil–liquid interface, and then the mixture was poured into a beaker filled with ultrapure water. (4) After n-hexane evaporated, the AgNP monolayer was transferred to the surface of supports, dried naturally, and the multilayer structure was prepared by performing layer-by-layer deposition. The prepared SERS substrates were thoroughly sequentially washed with ethanol and ultrapure water, and then dried with nitrogen.

Notably, to facilitate the description, the AgNP multilayers on the glass and Au film are denoted as *n*-AgNP@glass and *n*-AgNP@Au film, respectively, where *n* represents the number of layers of AgNPs, ranging from one to four.

### 2.4. Characterization of AgNP Multilayer SERS Substrates

The morphology of the AgNP multilayer SERS substrates was observed with a scanning electron microscope (JEOL JSM-7800F system, JEOL, Akishima, Japan). The ultraviolet–visible (UV–vis) extinction spectra of AgNP multilayer SERS substrates were observed with a UV-2450 spectrophotometer (Shimadzu, Kyoto, Japan). For *n*-AgNP@glass substrates, their extinction spectra were obtained by performing a transmission test, while those for *n*-AgNP@Au film substrates were converted through their reflection spectra.

### 2.5. SERS Measurements

R6G was selected as a reporter molecule for all SERS measurement experiments. The R6G powder was dissolved in ultrapure water to prepare a 10 times diluted concentration of 1 × 10^−15^–1 × 10^−7^ M. Prior to SERS measurements, the SERS substrates were immersed in the R6G aqueous solution for about 30 min to ensure uniform adsorption. SERS spectra were collected using a micro-Raman spectroscopy system (Horiba Jobin Yvon, HR Evolution, Paris, France) equipped with a 532 nm laser and a 50 × objective lens. All measurements were performed at room temperature. To ensure the reliability of the test results, the spectra of six random points were collected each of the specified times and averaged to obtain the final results.

## 3. Results and Discussion

### 3.1. Simulation Analysis

**Figure 1:**
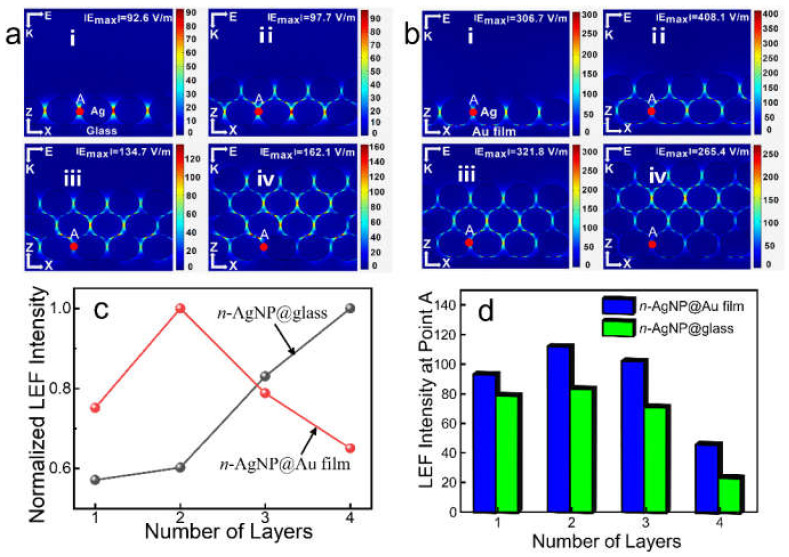
LEF distribution simulation results of (**a**) the *n*-AgNP@glass and (**b**) the *n*-AgNP@Au film. (**c**) Normalized maximum LEF intensity of the *n*-AgNP@glass and the *n*-AgNP@Au film. (**d**) The maximum LEF intensity histogram at point A of the *n*-AgNP@glass and the *n*-AgNP@Au film. *n* = 1, 2, 3, 4.

Figure 1a(i–iv) show the simulation results of LEF distribution of the *n*-AgNP@glass (*n* = 1, 2, 3, 4). Clearly, the number of LEF hotspots increased with the increase in AgNPs layers. Due to the surface plasmon hybridization between layers, the LEF intensity improved with the increase in AgNPs layers (Figure 1c), which is in agreement with the results reported in the literature [17,18]. For 1-AgNP@glass and 2-AgNP@glass, the maximum LEF hotspots were located at the gaps between adjacent AgNPs at the bottom layer. For 3-AgNP@glass and 4-AgNP@glass, the position of the maximum LEF hotspots has moved up, and were located at the gaps between adjacent AgNPs of Layer 1 and Layer 2 (here, the AgNPs layer’s serial numerals (1–4) increases from bottom to top for each model). As shown in Figure S3, when there were five layers of AgNPs on the glass support (5-AgNP@glass), the strongest LEF hotspots further shifted upward to the gaps between adjacent AgNPs of Layer 2 and Layer 3. It can be seen that the position of the strongest LEF hotspots of the *n*-AgNP@glass has a trend of gradually shifting upward as the layers of AgNPs increased. However, as can be seen from Figure 1d, the LEF intensity at point A (in the gaps between adjacent AgNPs at the bottom layer, which is marked in Figure S1) has a decreasing trend when the number of layers exceeds two, mainly because the scattering effect of nanoparticles led to the incident light flux gradually decreasing from the top to the bottom, and the plasma coupling effect of the bottom AgNPs weakening [15].

Figure 1b(i–iv) show the simulation results of LEF distribution of the *n*-AgNP@Au film (*n* = 1, 2, 3, 4). Similar to the *n*-AgNP@glass, the number of LEF hotspots in *n*-AgNP@Au film also increased with the increase in AgNPs layers. However, compared with the *n*-AgNP@glass, the *n*-AgNP@Au film generates many extra higher LEF “hotspots” at the gaps between Au film and AgNPs. Moreover, the maximum LEF of the *n*-AgNP@Au film always appeared in the gaps between Au film and AgNPs, which was much larger than that of the other layers. As shown in Figure 1c, the maximum LEF intensity was obtained when there were just two layers of AgNPs (2-AgNP@Au film), while the LEF intensity began to decrease with the further increase in the AgNPs layer (3-AgNP@Au film and 4-AgNP@Au film). In addition, the simulation results show that the maximum LEF intensity of the 2-AgNP@Au film (~408.1 V/m) was much higher than that of the 4-AgNP@glass (~162.1 V/m), which indicated that the 2-AgNP@Au film had a much higher SERS activity.

The above phenomena could be explained as follows: The Au film supports propagating surface plasmons (PSPs) [24,25], and the electric field associated with the PSPs is driven exclusively by the electric field due to the LSPR of the AgNPs and not directly by the incident radiation [26]. Moreover, the magnitude of the PSP-related field is proportional to the local field intensity of the AgNPs [26]. When the AgNP was placed on the Au film, its PSPs was able to hybridize with the local surface plasmon resonance (LSPR) of the AgNPs, resulting in plasmon gap modes and the generation of considerable LEF enhancement [2,13,27,28,29]. However, the LSPR of the AgNPs would decrease significantly with the increase in the distance [30,31]. Therefore, the LSPR of the bottom AgNPs closely adjacent to the Au film plays a leading role in the excitation of PSPs [32], which would greatly affect the LEF intensity at the gaps between the AgNPs and the Au film. As discussed above, the bottom-layer AgNPs had the largest LEF intensity in 2-AgNP@glass. When the glass support was replaced by the Au film, the LSPR of the bottom layer AgNPs could hybridize with the PSPs of the Au film, resulting in a significant enhancement of the LEF in these regions. Therefore, the 2-AgNP@Au film had a strongest LEF intensity. This was an optimal discovery: on the one hand, the manufacturing of an SERS substrate with fewer AgNPs layers is much easier; thus, it usually means more it is cost-effective; on the other hand, the SERS substrate with only two-layer of AgNPs avoids the problem that it is difficult for target molecules to enter the hotspots at the bottom layer due to the steric hindrance [18].

### 3.2. Characterization of the SERS Substrates

**Figure 2:**
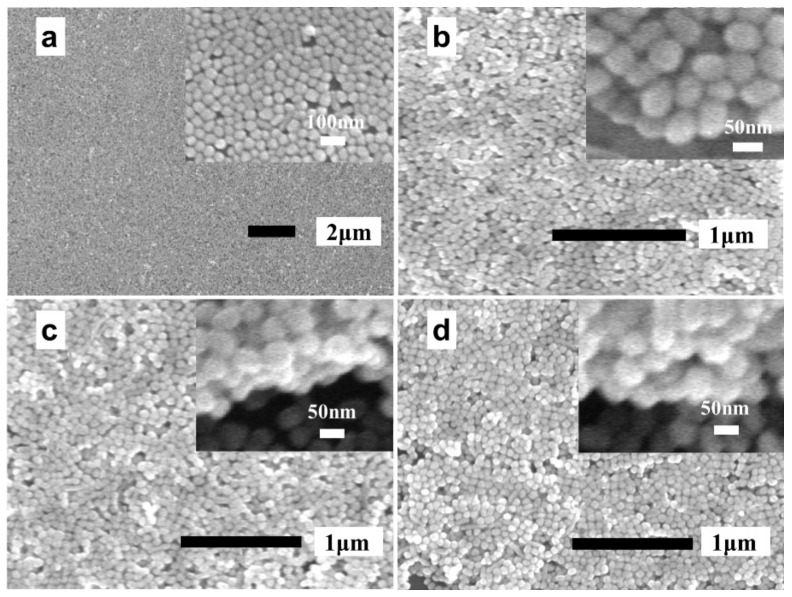
(**a**–**d**) SEM images of the *n*-AgNP@Au film (*n* = 1, 2, 3, 4).

Figure 2a–d show the scanning electron microscopy (SEM) images of one to four layers of AgNPs, in which the upper right corner insets of each figure is a local enlarged image. As displayed in Figure 2a, the prepared AgNP monolayer exhibits good uniformity in general, although a few stacked AgNPs and voids were also observed. However, it is inevitable that certain AgNP layers will be affected by the previously assembled AgNPs layers, i.e., the surface of the support is no longer flat, leading to a reduced uniformity of the AgNPs layers assembled later on in the procedure. Obviously, this phenomenon would actually worsen with the increase in layers. As illustrated in the upper right corner of Figure 2b–d, the hierarchical structures of the samples were evident, which indicated the successful preparation of the AgNP multilayers.

**Figure 3:**
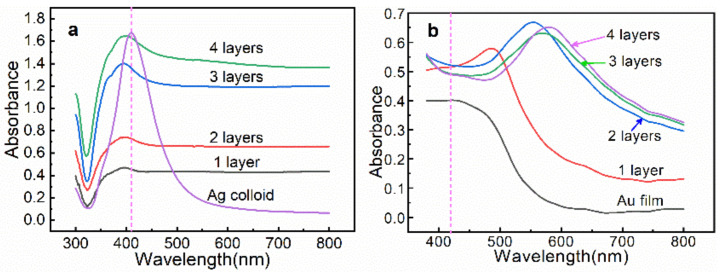
(**a**) UV–vis extinction spectra of the Ag colloids and the *n*-AgNP@glass substrates. (**b**) UV–vis extinction spectra of the Au film and the *n*-AgNP@Au film substrates.

Figure 3a shows the UV–vis extinction spectra of the Ag colloids and the *n*-AgNP@glass substrate. The optical response of the Ag colloids in water exhibits a plasmonic band centered at 415 nm. Compared with the Ag colloids, the maximum plasmonic peak of the *n*-AgNP@glass was not only blue-shifted because the average refractive index of air was lower than that of water, but its spectral bandwidth was also significantly broadened. Meanwhile, the extinction increased along with the increase in the layers, indicating that the flux of incident light decayed exponentially along the film stacking direction as the number of layers increased [15]. Figure 3b shows the UV–vis extinction spectra of the blank Au film and *n*-AgNP@Au film, in which the vertical imaginary line indicated the maximum plasmonic peak of the Ag colloids. Compared with the Ag colloids, the *n*-AgNP@Au film displayed a broad and red-shifted extinction as the layers increased. The optical response indicated the formation of AgNP assemblies on Au film, which caused intense plasmon coupling among particles and the Au film, and, therefore, LEF hotspots. In particular, 2-AgNP@Au film exhibited a maximum extinction under the excitation wavelength of 532 nm, which yielded a stronger electromagnetic field coupling with the excitation laser, thus leading to a better SERS efficiency. Encouragingly, this was consistent with our previous simulation results.

### 3.3. Optimization of SERS Substrates

**Figure 4:**
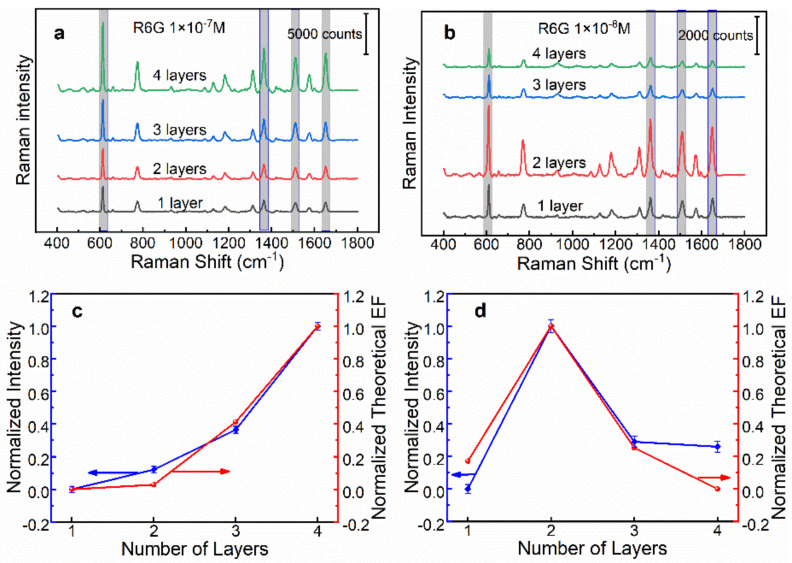
(**a**) The SERS spectra of 1 × 10^−7^ M R6G collected on the *n*-AgNP@glass substrate (*n* = 1, 2, 3, 4). (**b**) The SERS spectra of 1 × 10^−8^ M R6G collected on the *n*-AgNP@Au film substrate (*n* = 1, 2, 3, 4). (**c**,**d**) The variation of normalized SERS intensity at 612 cm^−1^ and normalized theoretical enhancement factor with the layer number of AgNPs on glass and Au film supports, respectively.

To gain insight into the SERS performance of these two types of SERS substrates, we have studied the overall efficiency in terms of the number of layers of AgNPs. SERS measurement conditions were set as follows: laser power 0.5 mW, integration time 2 s. Figure 4a shows the SERS spectra of 1 × 10^−7^ M R6G collected on the *n*-AgNP@glass substrates. As clearly shown, the Raman intensity at representative peaks (such as 612, 1308, 1358, and 1509 cm^−1^) increased with the increase in the number of layers. This was predicted from the optical simulation, revealing that the *n*-AgNP@glass substrate showed stronger plasmonic coupling deriving from more closely stacked AgNPs. Figure 4b displays the SERS spectra of 1 × 10^−8^ M R6G collected on the *n*-AgNP@Au film substrates. A marked increase in the Raman intensity was observed by moving from one to two layers of AgNPs, whereas it dropped sharply as the layers continued to increase. These results were also in agreement with the simulation results and optical properties.

For the purpose of visual comparison, we plotted the normalized average SERS intensity at 612 cm^−1^ and the normalized theoretical enhancement factor as a function of the layer number of AgNPs. As illustrated in Figure 4c,d, the experimental results have a similar trend with the theoretical simulation results, which further verified the correctness of the theoretical simulation. Overall, the above results showed that for both supports, i.e., the glass and Au film, 4-AgNP@glass and 2-AgNP@Au film have the best SERS efficiency.

### 3.4. Assessment of the SERS Performance

**Figure 5:**
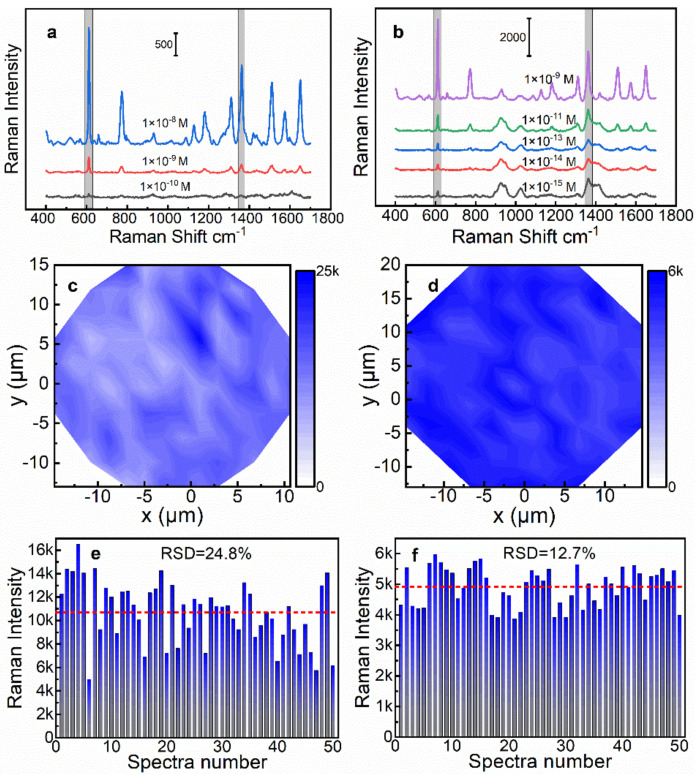
SERS spectra for different concentrations of R6G on (**a**) 4-AgNP@glass SERS substrate and (**b**) 2-AgNP@Au film SERS substrate. SERS mapping results of the 4-AgNP@glass SERS substrate (**c**) and the 2-AgNP@Au film SERS substrate (**d**). Different batch repeatability test results of (**e**) the 4-AgNP@glass SERS substrate and (**f**) the 2-AgNP@Au film SERS substrate.

As mentioned, the 4-AgNP@glass and the 2-AgNP@Au film had optimal SERS efficiency for the two different supports. Next, SERS measurements were performed using gradient concentrations (1 × 10^−8^~1 × 10^−15^ M) of R6G molecules to evaluate the SERS activity of both SERS substrates. SERS measurement conditions: laser power 0.5 mW, integration time 3 s. The detailed SERS spectra of R6G are illustrated in Figure 5a,b, and the characteristic Raman peaks at 612, 1308, 1358, and 1509 cm^−1^ were clearly observed in the spectra, while the Raman intensities gradually decreased with a decrease in the R6G concentration. For the 4-AgNP@glass substrate, the lowest concentration that can detect the signal was 1 × 10^−10^ M. However, for the 2-AgNP@Au film, the Raman peak at 612 cm^−1^ remained clear even down to 1 × 10^−15^ M, which indicated that the latter had a higher sensitivity.

The SERS performance of the 4-AgNP@glass and the 2-AgNP@Au film was finally quantified by estimating the analytical enhancement factor (AEF), which could be defined as follows [5]:

(1) $$\mathrm{AEF}=\frac{I_{SERS}/C_{SERS}}{I_{RS}/C_{RS}}$$

where *I_RS_* is the Raman intensity from an analyte solution with a concentration of *C_RS_* under non-SERS conditions, and *I_SERS_* is the Raman intensity from the same analyte on an SERS substrate, with a possibly different concentration (*C*_SERS_). Figure S4 illustrated the Raman spectra of the R6G molecules recorded on the glass slide, 4-AgNP@glass substrate, and 2-AgNP@Au film substrate under the same experimental conditions. As a result, the AEF of the 4-AgNP@glass substrate and the 2-AgNP@Au film substrate were calculated to be ~8.8 × 10^6^ and ~2.5 × 10^10^, respectively. The calculation results showed that the SERS activity of the 2-AgNP@Au film substrate was much stronger than that of the 4-AgNP@glass substrate. Significantly, compared with the reported SERS substrate [16,33,34,35,36,37,38], the 2-AgNP@Au film substrate showed excellent SERS activity. Figure S5 (Supplementary Materials) shows the fitting curve of the logarithmic concentration of R6G (10^−9^ M~10^−15^ M) and the logarithmic SERS intensity at 612 cm^−1^. The formula of fitting curve is: y =0.02+0.77x +8.39, and the correlation coefficient is 0.94. The result indicates that the 2-AgNP@Au film substrate has great potential for quantitative SERS measurements.

The signal uniformity is an important indicator to evaluate the performance of an SERS substrate. Thus, we further characterized the signal uniformity of the above two SERS substrates by using the SERS mapping technique. An elliptical region was randomly selected on the SERS substrate for the SERS mapping measurement (Figure S6a,b, Supplementary Materials). The R6G solution with a concentration of 1 × 10^−8^ M was used for the SERS mapping measurement of the 2-AgNP@Au film substrate. However, in order to obtain a stronger signal, we selected a higher concentration of R6G solution (1 × 10^−7^ M) for the same experiment of the 4-AgNP@glass substrate. The SERS mapping images were drawn based on the Raman intensity at 612 cm^−1^ of the R6G, as illustrated in Figure 5c,d. It is clear that the color distribution of the SERS mapping image of the 2-AgNP@Au film substrate was more uniform than that of the 4-AgNP@glass substrate, indicating that the 2-AgNP@Au film substrate had a better signal uniformity. For the 4-AgNP@glass substrate, the relative standard deviation (RSD) of the Raman intensity at 612 cm^−1^ was calculated to be 22.3%, while it was 11.0% for the 2-AgNP@Au film substrate. The main reason was that the distribution of the AgNPs was worse with the increase in the layers, which was confirmed by SEM imaging (Figure 2).

The reproducibility is another important indicator to evaluate the performance of the SERS substrate. Therefore, we prepared five different batches of 4-AgNP@glass substrates and 2-AgNP@Au film substrates, respectively, by using the same approaches described above. Then, the SERS spectra of R6G on 10 random points were collected for each substrate under the same test conditions. As illustrated in Figure 5e,f, we plotted the histogram of Raman intensities at 612 cm^−1^ of 4-AgNP@glass substrates and 2-AgNP@Au film substrates, respectively. As a result, the calculated RSD of the Raman intensity at 612 cm^−1^ was 24.8% for the 4-AgNP@glass substrates, while it was 12.7% for the 2-AgNP@Au film substrates. The above results indicated that the 2-AgNP@Au film substrate had a better reproducibility, which was consistent with our previous prediction, i.e., the fewer layers of AgNPs, the more controllable the quality of the SERS substrate.

## 4. Conclusions

In summary, we systematically studied the LEF distribution and characteristics of AgNP multilayers on the two supports, i.e., glass and Au film, through finite element simulations. The results showed that the maximum LEF could be obtained simply by depositing two layers of AgNPs on the Au film, which was much stronger than the optimal value on the glass. Then, one to four layers of AgNPs were successfully prepared on both supports, and the SERS experiments results were in good agreement with the simulations. Finally, the optimized SERS substrate, i.e., the 2-AgNP@Au film could realize the detection of R6G with 1 × 10^−15^ M, and the RSD of the Raman intensity was 11% and 12.7% for the same batch and different batches, respectively. In follow-up research, we intend to make further improvements to the nanoparticles distribution uniformity by optimizing the liquid–liquid interface self-assembly process to improve the reproducibility. Importantly, compared with existing nanoparticle multilayer SERS substrates—because only a nanoparticle bilayer needed to be prepared—the difficulty in terms of SERS substrate preparation was reduced, the steric hindrance on the molecules entering the bottom hotspots was weakened, and the influence of the incident laser and the underlying scattering signal being blocked or scattered by upper nanoparticle layers was diminished. Our work provides important theoretical guidance and a technical basis for the optimized design and preparation of high-performance SERS substrates.

## Data Availability

The data that support the findings of this study are available within the article.

## Supplementary Materials

The following supporting information can be downloaded at: https://www.mdpi.com/article/10.3390/bios13010052/s1, Figure S1: Simulation model of the various nanostructures; Figure S2: (a) SEM image of self-assembly of the AgNP monolayer. (b) The particle size distribution of the AgNPs. (c) The gap size distribution of adjacent AgNPs. Figure S3: LEF distribution of (a) the 5-AgNP@glass and (b) the 5-AgNP@Au film. Figure S4: Raman spectra of different concentrations of R6G on the 4-AgNP@glass substrate, 2-AgNP@Au film substrate, and glass substrate. Figure: S5 Microscopic images of the SERS mapping area of (a) the 4-AgNP@glass SERS substrate and (b) the 2-AgNP@Au film SERS substrate. Figure S6: Microscopic images of the SERS mapping area of (a) the 4-AgNP@glass SERS substrate and (b) the 2-AgNP@Au film SERS substrate.

## Abbreviations

AgNP	Ag nanoparticle
AEF	Analytical enhancement factor
EF	Enhancement factors
LSPR	Local surface plasmon resonance
UV–vis	Ultraviolet–visible
LEF	Local electric field
PSPs	Propagating surface plasmons
R6G	Rhodamine 6G
RSD	Relative standard deviation
SEM	Scanning electron microscopy
SERS	Surface-enhanced Raman spectroscopy
