# Supplementary material for: Optimized Design and Preparation of Ag Nanoparticle Multilayer SERS Substrates with Excellent Sensing Performance
Source: Biosensors (Basel). 2022 Dec 29;13(1):52. doi: 10.3390/bios13010052 (PMC9855442; doi:10.3390/bios13010052)
Supplement: Supplementary file 1 [file biosensors-13-00052-s001.zip › biosensors-2108998-supplementary.pdf]

biosensors

Supplementary Material

Optimized Design and Preparation of Ag Nanoparticle Multilayer SERS Substrates with Excellent Sensing Performance

Ping Wen, Feng Yang, Xiaoling Hu, Yi Xu , Shu Wan and Li Chen

1. Model of Simulation

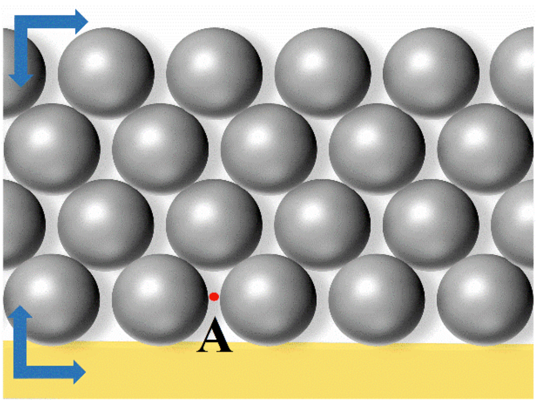

Figure S1. Simulation model of the various nanostructures.

2. Characterization of AgNP Monolayer

Figure S2a shows the scanning electron microscopy (SEM) image of the AgNP monolayer. The images show that the nanoparticles were roughly spherical. As shown in Figure S2b the average diameter of the AgNPs was about 50 nm, and the standard deviation of the diameters of the particles was 6.3 nm. These results demonstrated the low polydispersity of the size distribution, which provided a guarantee for the preparation of uniform SERS substrates. The average gap size between the particles was about 4.3 nm (Figures S2c).

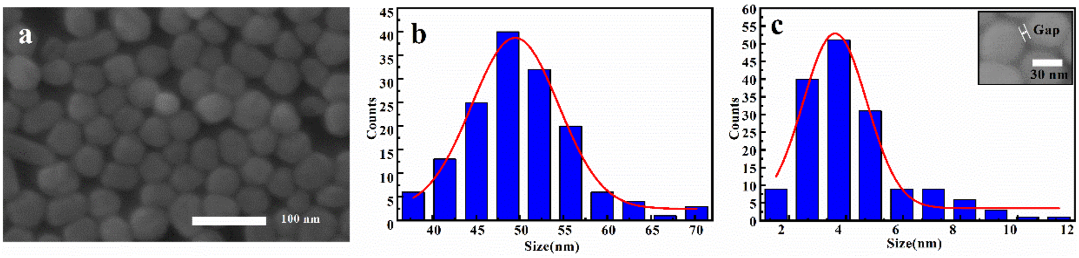

Figure S2. (**a**) SEM image of self-assembly of the AgNP monolayer. (**b**) The particle size distribution of the AgNPs. (**c**) The gap size distribution of adjacent AgNPs.

3. LEF Distribution of the 5-AgNP@Glass and the 5-AgNP@Au Film

Figure S3 shows the LEF distribution simulation results of the 5-AgNP@glass and 5-AgNP@Au film. For the 5-AgNP@glass, the strongest LEF hotspots located at the gaps between adjacent AgNPs of Layer 2 and Layer 3. For the 5-AgNP@Au film, the strongest LEF hotspots located at the gaps between the AgNPs and the Au film.

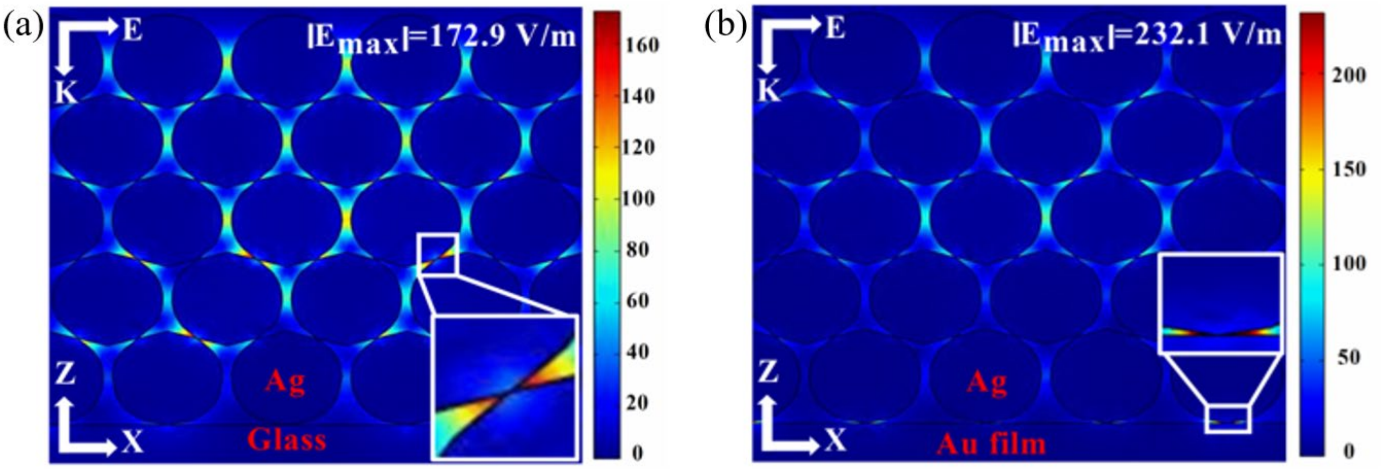

Figure S3. LEF distribution of (**a**) the 5-AgNP@glass and (**b**) the 5-AgNP@Au film.

4. Calculation of the Analytical Enhancement Factor

Figure S4 shows the Raman spectra of R6G collected on the glass substrate, 4-AgNP@glass substrate, and 2-AgNP@Au film substrate. The I_{RS} is Raman intensity from R6G solution with a concentration of 10^{-2} M(C_{RS}) under glass substrate. I_{SERS} is the Raman intensity from the R6G on 2-AgNP@Au film substrate with a concentration of 10^{-13} M(C_{SERS}) or 4-AgNP@glass substrate with a concentration of 10^{-9} M(C_{SERS}). The calculation results showed that the SERS enhancement factors of the 4-AgNP@glass substrate and the 2-AgNP@Au film substrate were about 8.8×10^6 and 2.5×10^{10}, respectively.

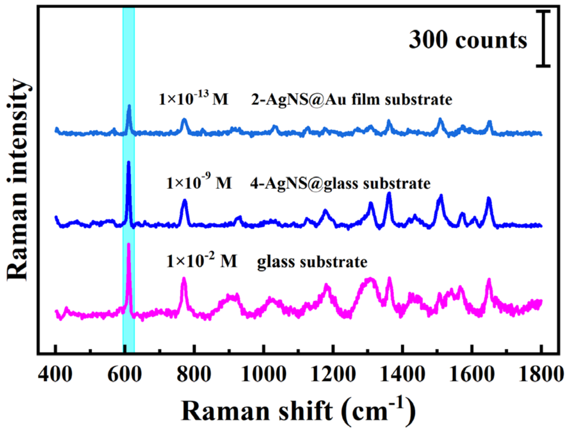

Figure S4. Raman spectra of different concentrations of R6G on the 4-AgNP@glass substrate, 2-AgNP@Au film substrate, and glass substrate.

5. The Evaluation on Quantitative Performance of 2-AgNP@Au Film

Figure S5 is the fitting curve of the logarithmic concentration of R6G (10^{-9} M~10^{-15} M) and the logarithmic SERS intensity at 612 cm^{-1}. The formula of fitting curve is: $y=0.02x^2+0.77x+8.39$, and the correlation coefficient is 0.94. The result indicates that the 2-AgNP@Au film substrate has great potential for quantitative SERS measurements.

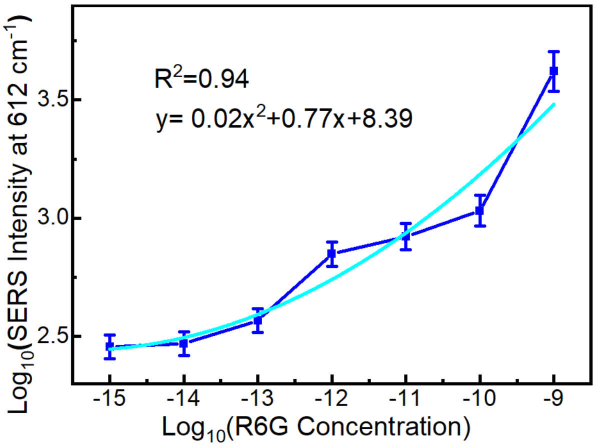

Figure S5. The logarithmic concentrations of R6G versus the logarithmic SERS intensity at 612 cm^{-1}. The error bars are the RSD obtained from five independent measurements.

6. SERS Mapping Area of the SERS Substrates

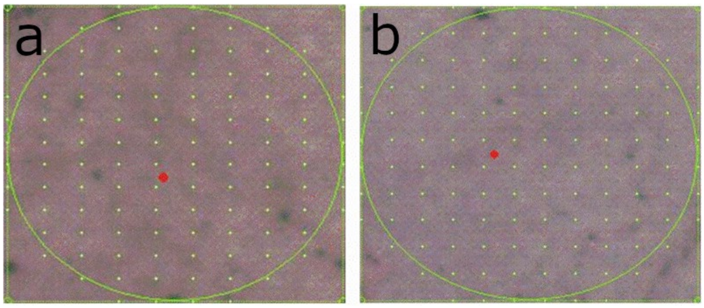

Figure S6. Microscopic images of the SERS mapping area of (**a**) the 4-AgNP@glass SERS substrate and (**b**) the 2-AgNP@Au film SERS substrate.
